# Psychometric properties of the Korean version of the Pittsburgh Fatigability Scale in breast cancer survivors

**DOI:** 10.1186/s12955-021-01815-8

**Published:** 2021-07-12

**Authors:** Min Kyeong Jang, Sue Kim, Chang Gi Park, Eileen G. Collins, Lauretta T. Quinn, Nancy W. Glynn, Carol Estwing Ferrans

**Affiliations:** 1 Department of Biobehavioral Nursing Science, College of Nursing, University of Illinois at Chicago, Chicago, IL USA; 2 University of Illinois Cancer Center, Chicago, IL USA; 3 College of Nursing, Mo-Im Kim Nursing Research Institute, Yonsei University, 50-1 Yonsei-ro, Seodaemun-gu, Seoul, 03722 Korea; 4 Department of Epidemiology, Graduate School of Public Health, University of Pittsburgh, Pittsburgh, PA USA

**Keywords:** Breast neoplasms, Cancer, Fatigability, Fatigue, Oncology, Psycho-oncology, Survivors, Validation

## Abstract

**Background:**

Fatigability has recently emerged in oncology as a concept that anchors patients’ perceptions of fatigue to defined activities of specified duration and intensity. This study aimed to examine the psychometric properties of the Korean version of the Pittsburgh Fatigability Scale (K-PFS) for women with breast cancer.

**Methods:**

This cross-sectional study involved 196 women with breast cancer recruited from a tertiary hospital in Seoul, Korea. Reliability was evaluated using Cronbach’s alpha, and confirmatory factor analysis was conducted to examine the factor structure of the K-PFS. Four goodness-of-fit values were evaluated: (1) the comparative fit index (CFI), (2) the Tucker–Lewis index (TLI), (3) the root mean square error of approximation (RMSEA), and (4) the standardized root mean square residual (SRMR).

**Results:**

Of the 196 survivors, 71.1% had greater physical fatigability (K-PFS Physical score ≥ 15) and 52.6% had greater mental fatigability (K-PFS Mental score ≥ 13). The Cronbach’s alpha coefficient for the total K-PFS scale was 0.926, and the coefficients for the physical and mental fatigability domains were 0.870 and 0.864, respectively. In the confirmatory factor analysis for physical fatigability, the SRMR value (0.076) supported goodness of fit, but other model fit statistics did not (CFI = 0.888, TLI = 0.826, and RMSEA = 0.224). For mental fatigability, although three goodness-of-fit values were acceptable (CFI = 0.948, TLI = 0.919, and SRMR = 0.057), the RMSEA value (0.149) did not indicate good model fit. However, each item coefficient was statistically significant (> 0.5), and the K-PFS was therefore found to be valid from a theoretical perspective.

**Conclusion:**

This study provides meaningful information on the reliability and validity of the K-PFS instrument, which was developed to meet an important need in the context of breast cancer survivors. Additional research should examine its test–retest reliability and construct validity with performance measures.

## Background

Fatigue, which is one of the main issues faced by cancer survivors, is a predictor of morbidity and mortality in survivors of various types of cancer [1–3]. The severity of fatigue is known to be affected by multiple factors, including the cancer itself and the type of cancer treatment. Furthermore, the prevalence of fatigue varies during survivorship, and cancer survivors can experience short-term and long-term fatigue even after they complete treatment [4–6]. Thus, it is important to be able to accurately measure fatigue levels and the ability to overcome fatigue in cancer survivors. Fatigue can be defined as an individual’s perceived lack of physical and/or mental vitality; it is therefore subjective in nature and should be measured as such [7]. However, the definitions of fatigue differ considerably, as is reflected by the varying terminology currently used to refer to fatigue [7]. In addition, fatigue measures have self-pacing bias because they do not anchor perceived fatigue to activity intensity and duration [8]. Furthermore, even if the concept of fatigue is consistently defined and applied, healthcare providers may be unable to measure significant differences in fatigue over time because the ability to overcome fatigue varies among individual survivors.

To improve upon the limitations imposed by fatigue-related methodological issues, and to accurately measure individuals’ potential ability to overcome the physical and mental dimensions of fatigue, the concept of fatigability has recently emerged. Distinct from the concept of fatigue, fatigability refers to the relationship between the individual’s subjectively measured perceptions of fatigue and various types and levels of objectively measured activity [9]. In other words, fatigability refers to how readily individuals feel physically and mentally fatigued, rather than centering on the concept of fatigue itself. For research purposes, fatigability can be measured by combining self-reported fatigue with quantified physical or cognitive activity, provided that the effort associated with the activity can be standardized by intensity and duration. The measurement advantages offered by the concept of fatigability allow meaningful comparisons across participants and studies.

Fatigability has been applied in various areas of research, including studies of older adults and patients affected by neurologic illnesses and pulmonary disease [7–10], and in recent years, this concept has also been applied in studies of cancer patients [11–14]. Three categories of fatigability have been established for different measurement purposes, including self-reported, perceived, and performance fatigability [8, 15]. As a measurement tool for fatigability, the Pittsburgh Fatigability Scale (PFS) is a valid and reliable measure of perceived fatigability that can be used to achieve alignment with performance fatigability [16]. The PFS was originally developed with consideration of various levels of metabolic equivalents (METs), which describe the intensity of tasks. The PFS includes four activity categories corresponding to MET levels: social, sedentary (≤ 1.5 METs), lifestyle or light-intensity (1.6–2.9 METs), and moderate to high-intensity (≥ 3 METs). Fatigability should be assessed in cancer survivors because it is important to understand their subjectively measured perceptions of fatigue and how they are related to objectively measured activity. Another issue is that intervention studies’ findings may be masked because of the methodological problems with measuring fatigue. Thus, the PFS can be easily applied to assess both physical and mental fatigability in cancer survivors.

No fatigability measures are available for use in South Korea. To meet the need for such a measure, the PFS was translated into Korean in 2018. However, no psychometric evaluation of the Korean version of the PFS (K-PFS) has been performed. Given the importance of the concept of fatigability and the major problems that fatigue poses for cancer survivors, psychometric testing of the K-PFS is urgently needed so that the instrument can be applied in Korean research and practice. Thus, the purpose of this study was to evaluate the psychometric properties of the K-PFS. The study had two specific aims: to evaluate the internal consistency of the K-PFS and to assess its validity through confirmatory factor analysis.

## Methods

### Design

This study employed a cross-sectional research design with convenience sampling to evaluate the psychometric properties of the K-PFS.

### Setting and sample

Following approval by the Institutional Review Board (#4-2018-0386), a convenience sample of 196 women with breast cancer was recruited from Yonsei Medical Center in Seoul, Korea; the sample included both clinic patients and members of four support groups. First, women with breast cancer were recruited when they visited the breast cancer clinic of the medical center. Clinic staff introduced the study to eligible women with breast cancer, and interested women were introduced to the principal investigator (PI) to discuss the research. Second, women with breast cancer also were recruited from hospital-based breast cancer support groups. At support group meetings, the PI described the study and met with group members who were interested in participating. In both circumstances, the PI confirmed women’s eligibility, obtained their written informed consent, and asked them to complete the study questionnaire in a private conference room. No information was provided by the clinic or support group leaders about women who chose not to talk to the PI. A total of 199 women with breast cancer spoke with the PI about study participation, but three women did not have time to complete the survey due to medical appointments. Thus, data were collected from 196 breast cancer survivors and analyzed. The target sample size for factor analysis was considered to be 5 to 10 participants per item, and confirmatory factor analysis required between 130 and 200 participants [17]. Considering that the K-PFS consists of 10 items each for physical and mental fatigability, 196 was a sufficient sample size for the purposes of this study. The participants included in this study met the following inclusion criteria: they (1) were more than 20 years old, (2) were Korean women, (3) had been diagnosed with breast cancer at least 1 year previously, and (4) had completed active cancer treatment (radiotherapy and/or chemotherapy).

### Instrument description

Glynn and colleagues published the 10-item PFS using factor analysis with varimax rotation [16] and recently Renner and colleague validated the mental PFS [18]. In our study, fatigability was measured using the K-PFS, which had been translated from the PFS with the participation of Glynn and colleagues and subjected to cognitive interview-based assessment [19]. The PFS is intended to assess physical and mental fatigability with respect to fatigue level in relation to intensity and duration of activity. The PFS is a self-report instrument designed to measure four categories of fatigability: social (2 items), sedentary (2 items), lifestyle or light intensity (2 items), and moderate to high intensity (4 items). The 10-item instrument assesses the levels of physical and mental fatigue that respondents expect to feel immediately after various activities. Each item is rated on a scale ranging from 0 (no fatigue) to 5 (extreme fatigue), and possible total scores range from 0 to 50; higher scores indicate greater physical and mental fatigability. The PFS Physical score of 15 or above indicated a greater level of physical fatigability, and the PFS Mental score of 13 or above was identified as indicating a greater level of mental fatigability [20–22]. In a previous study, the instrument was found to have high concurrent and convergent validity in relation to performance fatigability, mobility, physical function, and fitness, and its internal consistency was good, with a Cronbach’s alpha for fatigability of 0.88 [16]. Also, a recent study showed a good internal consistency for perceived mental fatigability (Cronbach’s alpha = 0.85) [18].

### Translation process

The translation process for the K-PFS was guided by a linguistic validation manual for health outcome assessments [23]. This process included forward- and back-translation and committee review of the Korean-language cross-cultural adaptation of the instrument. This translation effort was conducted by three authors of this paper. English-to-Korean translation was carried out by four bilingual researchers in collaboration with the original PFS developer. At each stage of the process, all translation issues were discussed and reconciled. Following translation, four members of the general population and 16 breast cancer survivors participated in cognitive interviews regarding the instrument. After the research team reached agreement on the final language of the K-PFS, the instrument was administered to 196 Korean breast cancer survivors. The final version of the K-PFS is available from Dr. Nancy W. Glynn upon request [https://publichealth.pitt.edu/epidemiology/research-practice/faculty-research/pittsburgh-fatigability-scale].

### Data collection

All data were collected cross-sectionally from June 2018 to September 2018. The demographic and clinical questionnaire was developed by the research team as a structured self-report data collection tool. The K-PFS questionnaire was used to assess the severity of fatigability, and its psychometric properties were evaluated. Participants were asked to complete the self-administered questionnaires either at the outpatient clinic or support group meetings, and completing the questionnaire took about 60 min.

### Data analysis for psychometric assessment

Both Stata (version 16) and SPSS (version 25) were used for data analysis. The participants’ general and disease-related characteristics were analyzed using descriptive statistics. Our research team followed the original developer’s imputation instructions for missing physical and mental fatigability data. The reliability of the K-PFS was estimated by internal consistency (Cronbach’s α). Reliability coefficients were calculated for both physical and mental fatigability subscales, as well as for the entire instrument by analyzing all 20 items as a single scale. A coefficient of 0.70 was employed as an accepted standard of minimum reliability [24]. In addition, confirmatory factor analysis was conducted to examine the factor structure of the K-PFS. Employing MPlus 8, this analysis was performed using an ordinal scale and a robust weighted least square mean and variance–adjusted estimator. Four goodness-of-fit values were assessed: (1) the comparative fit index (CFI), (2) the Tucker–Lewis index (TLI), (3) the root mean square error of approximation (RMSEA), and (4) the standardized root mean square residual (SRMR). Higher CFI and TLI values indicate better model fit, with values of 0.95 or more indicating good model fit and values above and near 0.90 indicating acceptable fit [25]. RMSEA values below 0.06 indicate excellent model fit, while values exceeding this criterion provide inadequate support for good fit. Lastly, SRMR values lower than 0.08 indicate good model fit [25].

## Results

### General and disease-related characteristics of participants

The demographic and clinical characteristics of participants are presented in Table 1. The average age of the 196 women with breast cancer was 55.4 years, with a range of 29 to 76 years. Over two-thirds of the participants (164, 83.7%) were married, and approximately 85% of the participants had stage I or II breast cancer. The average time since breast cancer diagnosis was 7.06 years, with a range from 1.05 to 21.56 years. Over two-thirds of the participants (n = 132, 70.6%) had received radiation therapy, and over three-quarters (n = 140, 76.1%) had received chemotherapy.

**Table 1:** Demographic and clinical characteristics (*N* = 196)

Characteristics	Mean ± SD (Range)		*n*	%	
Age (years)	55.4 ± 8.7 (29–76)				
Marital status					
Single			9	5.0	
Married			164	83.7	
Widowed			16	8.2	
Divorced/separated			7	3.6	
Education					
≤ High school graduate			106	54.1	
College graduate			77	39.3	
≥ Graduate degree			13	6.63	
Employment					
Full-time			44	22.4	
Part-time			12	6.1	
Not employed			108	55.1	
Religion					
Yes			141	71.9	
No			55	28.1	
Cancer stage					
Stage I			91	46.7	
Stage II			77	39.5	
Stage III			25	12.8	
Stage IV			2	1.03	
Operation type					
Breast-conserving surgery			88	44.9	
Mastectomy			108	55.1	
Time since diagnosis (years)	7.1 ± 4.6 (1.1–21.6)				
Cancer Treatment					
Chemo-radiation			106	57.6	
Chemotherapy only			34	18.5	
Radiation therapy only			26	14.1	
Surgery only			18	9.8	
K-PFS: Korean-Pittsburgh Fatigability Scale					
K-PFS Physical score	20.5 ± 9.1 (2–47)				
K-PFS Mental score	14.4 ± 9.6 (0–50)				

### Breast cancer survivors’ fatigability

The mean fatigability scores, standard deviations, and score ranges among participants are shown in Table 2. The mean PFS Physical score was 20.5 (SD = 9.1) and the mean PFS Mental score was 14.4 (SD = 9.6). Among the items addressing physical fatigability, the highest item response (M = 3.24, SD = 1.43) was seen for the item addressing 30 min of moderate- to high-intensity strength training, indicating that women felt greater susceptibility to fatigue when they attempted muscle strengthening exercises. The highest mental fatigability item response (M = 2.53, SD = 1.61) was observed for the item involving hosting a 1-h social event, suggesting that women found it mentally wearisome to take a lead role in social encounters.

**Table 2:** Mean scores of physical and mental fatigability using the Korean-Pittsburgh Fatigability Scale (*N* = 196)

Subdomains	Item	Description	Physical		Mental	
			Mean	SD	Mean	SD
Social activity	8	Participating in a social activity for 1 h	1.82	1.40	1.58	1.51
	9	Hosting a social event for 1 h	2.31	1.48	2.53	1.61
Sedentary activity	5	Watching TV for 2 h	1.05	1.28	0.81	1.22
	6	Sitting quietly for 1 h	0.79	1.11	0.65	1.11
Lifestyle or light-intensity activity	1	Leisurely walk for 30 min	1.04	1.26	0.56	1.09
	3	Light household activity for 1 h	2.16	1.33	1.74	1.48
Moderate- to high-intensity activity	2	Brisk or fast walk for 1 h	2.44	1.41	1.08	1.43
	4	Heavy gardening or outdoor work for 1 h	2.48	1.32	1.51	1.47
	7	Moderate- to high-intensity strength training for 30 min	3.24	1.43	1.94	1.78
	10	High-intensity activity for 30 min	3.18	1.37	1.98	1.71

### Internal consistency reliability of the K-PFS

The overall Cronbach’s alpha coefficient for the K-PFS total scale was 0.926, indicating good internal consistency. The Cronbach’s alpha coefficients for the K-PFS physical and mental fatigability subscales were 0.870 and 0.864, respectively, also indicating good internal consistency. Regarding physical fatigability, the alpha coefficients for the four physical activity levels—social, sedentary, lifestyle or light-intensity, and moderate- to high-intensity activity—were 0.835, 0.843, 0.634, and 0.783, respectively. With respect to mental fatigability, the alpha coefficients were 0.737, 0.826, 0.539, and 0.807 for these four activity levels, respectively.

### Confirmatory factor analysis for the K-PFS

Regarding physical fatigability, confirmatory factor analysis of the four-factor model showed that two goodness-of-fit values (CFI = 0.888, TLI = 0.826) did not meet the recommended criteria. The RMSEA value (0.224) for physical fatigability also did not support good model fit. However, the SRMR value (0.076) for physical fatigability indicated that the fit was good. Regarding mental fatigability, the CFI (0.948), TLI (0.919), and SRMR (0.057) values indicated acceptable fit, but the RMSEA value (0.149) did not.

The standardized coefficient estimates between each item and the corresponding subdomains are shown in Table 3. The standardized coefficients for each factor and the corresponding items for physical fatigability were 0.836–0.921 for the social activity subdomain, 0.857–0.949 for the sedentary activity subdomain, 0.719–0.745 for the lifestyle or light-intensity activity subdomain, and 0.612–0.845 for the moderate- to high-intensity activity subdomain. All standardized coefficients were greater than 0.5, suggesting that each physical fatigability item is a valid indicator of the four factors. The standardized coefficients for each factor and the corresponding items for mental fatigability were 0.534–1.240 for the social activity subdomain, 0.845–0.959 for the sedentary activity subdomain, 0.617–0.794 for the lifestyle or light-intensity activity subdomain, and 0.726–0.861 for the moderate- to high-intensity activity subdomain. Except for item 8, the coefficient of which unexpectedly exceeded 1.0, the standardized coefficients for mental fatigability items were greater than 0.5 and less than 1.0.

**Table 3:** Confirmatory factor analysis of Korean-Pittsburgh Fatigability Scale

Subdomains	Items	Physical Fatigability			Mental Fatigability		
		Coef	SE	*p*	Coef	SE	*p*
Social activity	8	0.921	0.037	< .001	1.240	0.138	< .001
	9	0.836	0.035	< .001	0.534	0.070	< .001
Sedentary activity	5	0.949	0.035	< .001	0.959	0.027	< .001
	6	0.857	0.037	< .001	0.845	0.038	< .001
Lifestyle or light-intensity activity	1	0.745	0.043	< .001	0.794	0.058	< .001
	3	0.719	0.042	< .001	0.617	0.056	< .001
Moderate to high intensity activity	2	0.803	0.035	< .001	0.823	0.034	< .001
	4	0.845	0.033	< .001	0.861	0.030	< .001
	7	0.612	0.04	< .001	0.726	0.041	< .001
	10	0.742	0.034	< .001	0.769	0.035	< .001

## Discussion

The present study was performed to assess the psychometric properties of the K-PFS and was the first to examine both instrument reliability and validity with a specific focus on Korean women with breast cancer.

The internal consistency of the K-PFS was confirmed by item-total correlations; the internal consistency reliability for physical and mental fatigability was 0.87 and 0.86, respectively. Similarly, a previous psychometric study of the PFS involving older adults reported good internal consistency (Cronbach’s alpha = 0.88) and excellent test–retest reliability (intra-class correlation = 0.86) [16]. Also, a recent psychometric study of the PFS mental fatigability subscale showed good internal consistency (Cronbach’s alpha = 0.85) and good test–retest reliability (intra-class correlation = 0.78) [18]. Compared to the previous two studies [16, 18], the Cronbach’s alpha values for total physical and mental fatigability were also high in the present study, demonstrating that all items were closely correlated with the physical and mental fatigability subscales and had good internal consistency.

With respect to the four subdomains of physical and mental fatigability, three (social, sedentary, and moderate- to high-intensity activity) showed good internal consistency. Only the lifestyle or light-intensity activity subdomain showed weaker Cronbach’s alpha values (0.634 and 0.539, respectively). A low Cronbach’s alpha value can be related to a small number of items, poor inter-relatedness among items, or heterogeneous constructs [26]. The lifestyle or light-intensity activity subdomain had only two items—“leisurely walk for 30 min” (item 1) and “light household activity for 1 h” (item 3)—that were originally developed to represent METs between 1.6 and 2.9 [16]. Accordingly, the lower Cronbach’s alpha values may be attributable to the small number of items, as well as perceived differences in the activity types from the viewpoint of participants. Specifically, the mean scores for items 1 and 3 differed significantly. For both physical and mental fatigability, item 3 had higher mean scores (physical: 2.16 ± 1.33, mental: 1.74 ± 1.48) than item 1 (physical: 1.04 ± 1.26, mental: 0.56 ± 1.09) (t (− 11.68) = 195, *p* = 0.0000, t (− 11.09) = 194, *p* < 0.0001). However, since this subdomain was classified according to METs values, and the two items belong to the same METs class, the Cronbach’s alpha values alone do not necessarily translate into poor internal consistency. Because the PFS developers attempted to reflect METs as a theoretical factor, they included a variety of physical activity types corresponding to METs classes; however, when the PFS was developed, the 4 items in the original lifestyle or light-intensity activity subdomain were not included because they did not load as high [16]. Overall, the Cronbach’s alpha values for physical and mental fatigability indicate that the K-PFS has good reliability, and since the PFS encompasses various METs classes while allowing to look at the entire range of intensity, the K-PFS shows promise for use in both clinical practice and research. Further studies using the K-PFS are needed to assess other types of instrument reliability such as inter-rater, test–retest, and parallel-forms reliability.

With respect to instrument validity, the theoretical four-factor structure of the PFS was originally verified by means of factor analysis in 2015 [16]. This was done to ensure the consistency of the structure of item loadings with the originally hypothesized constructs. In the present study, the confirmatory factor analysis partially supported the four-subscale structure of the 10-item K-PFS. Specifically, the physical fatigability domain showed good model fit based on the SRMR value, but the other goodness-of-fit values were unacceptable. By way of comparison, in their original study, Glynn et al. (2015) established concurrent and convergent validity for the physical fatigability domain of the PFS using measures such as high perceived exertion, high performance deterioration, slow gait speed, worse physical function, and lower fitness. In addition, the researchers confirmed good overall discrimination of the PFS score for physical fatigability in comparison to performance measures with adjustments for age, sex, and race. In addition, in a recent study involving people aged over 60 years, Renner and colleagues validated the mental fatigability domain of the PFS [18]. Their confirmatory factor analysis with promax rotation using two factors (social and physical activities) supported a good model fit (SRMR = 0.064, RMSEA = 0.095, CFI = 0.91). Their assessment of concurrent and construct validity using global fatigue, depression, and cognition also showed moderate validity and their convergent validity was also strong. Consequently, similar to two previous studies reported a good model fit, our findings also partially indicated good model fit and the K-PFS was found to be valid from a theoretical perspective.

In a recent study that reported validation results for the Dutch PFS for older adults, the Dutch version had good content validity and construct validity, but confirmatory factor analysis of the original factor structure showed poor model fit (with SRMR and CFI values of 0.29 and 0.75, respectively) [27]. In another recent study involving people with and without chronic disease, convergent validity and discriminant validity were supported for the PFS [22]. Our findings partially support the validity of the K-PFS, but future studies should investigate its construct, discriminant, and criterion validity. In addition, because fatigability anchors perceptions of fatigue to defined activities of specified duration and intensity, comparisons with objective performance measures such as METs should be considered to assess the K-PFS’s validity.

This study highlights the severity of fatigability experienced by women with breast cancer and the importance of research on this problematic phenomenon. Our study showed that 71.1% of women had greater physical fatigability (M = 20.47, K-PFS Physical score ≥ 15) and that 52.6% had greater mental fatigability (M = 14.35, K-PFS Mental score ≥ 13). A previous study involving two generations of family members enriched for exceptional longevity and their spouses (*N* = 2355, M:73.7 years) using the same PFS instrument reported that physical fatigability prevalence was higher in older aged groups (e.g., 60–60 years: 28%, 90–108 years: 89.5%) [28], and another study involving 2,361 older adults (M:73.6 years) also showed that mental fatigability was strikingly greater with age (e.g., 60–60 years: 14.5%, 90–108 years: 67.2%) [22]. Compared to the previous two studies, we found it noteworthy that even after an average of more than 7 years, women with breast cancer (M:55.4 years) still experienced greater fatigability equivalent to very old adults. While the PFS has been mostly used with older adults, this study provides valuable data on Korean breast cancer survivors with the K-PFS, allowing for comparison with different clinical characteristics and various cultural settings. As to the PFS itself, ours was the first study to apply the instrument to Korean women with breast cancer. Consequently, in future studies, the PFS should be used to measure perceived physical and mental fatigability in women with breast cancer who have different clinical characteristics and in a variety of cultural settings.

Given that the average time following breast cancer diagnosis in this study was more than 7 years, our findings suggest that the K-PFS offers significant benefits for assessing and managing fatigability in women with breast cancer who are in various phases of treatment and survivorship. The PFS was initially developed for older adults, but has been found adequate for use in the general population [29], and the K-PFS shows similar potential. This is the case because when we translated the instrument from English to Korean in collaboration with the original developer of the PFS, we considered both the general population and women with breast cancer during our decision-making. For example, we modified some of the instrument’s examples of physical activity to reflect Korean culture, and we considered breast cancer survivors’ physical limitations as we selected examples to support respondents’ assessment of their level of fatigability. A previous study suggested that fatigability in older persons who are vulnerable to functional decline might serve as a predictor of impending decline in mobility [21]. From a similar viewpoint, because breast cancer survivors are likely to experience greater fatigability than older populations [20], and because various physical and psychological symptoms coexist in cancer patients [30–32], fatigability as measured by the K-PFS may also be an important predictor of mobility in patients with cancer. Accordingly, the K-PFS has the potential to be widely used for measuring fatigability among cancer survivors.

Several limitations of the present study need to be acknowledged. As the study employed a cross-sectional design, we could not assess test–retest validity for stability or other types of validity. As another limitation, the PFS was originally developed for the general population of the United States, but our psychometric evaluation of the K-PFS included only Korean breast cancer survivors. Although we culturally adapted the instrument for use with both the general population and women with breast cancer in Korea during the translation process, differences in the characteristics and health status of the research subjects involved in the PFS and K-PFS studies may partially explain inconsistencies in the psychometric findings. Also, because no previous study has applied the PFS to breast cancer survivors, no PFS psychometric data are available for other populations with similar clinical characteristics. Future validation studies should investigate the instrument’s psychometric properties both for general populations and for survivors of various other types of cancer as well as male cancer survivors.

## Conclusion

This study provides meaningful information on the reliability and validity of the K-PFS instrument, which was developed to meet an important need in the Korean context. The K-PFS is an easily administered instrument that can measure fatigability with respect to the intensity and duration of physical activities and overcomes the methodological concern of self-pacing evident in global fatigue measure. As we included culturally appropriate physical activity examples in the instrument and carefully chose examples of activity levels that would be relevant to Korean breast cancer survivors, the K-PFS appears to be suitable for use among cancer survivors, as well as in the general population of Korea. Future research should examine the test–retest reliability of the K-PFS and further evaluate its construct validity through the use of objective performance measures.

## Data Availability

The data set used and/or analyzed during the current study is available from the corresponding author on reasonable request.

## Abbreviations

CFI: Comparative fit index
PFS: Pittsburgh Fatigability Scale
K-PFS: Korean version of the Pittsburgh Fatigability Scale
METs: Metabolic equivalents
RMSEA: Root mean square error of approximation
SRMR: Standardized root mean square residual
TLI: Tucker–Lewis index
